# Regulatory Effects of Circular RNAs on Host Genes in Human Cancer

**DOI:** 10.3389/fonc.2020.586163

**Published:** 2021-02-11

**Authors:** Xiong Wang, Huijun Li, Yanjun Lu, Liming Cheng

**Affiliations:** Department of Laboratory Medicine, Tongji Hospital, Tongji Medical College of Huazhong University of Science and Technology, Wuhan, China

**Keywords:** circRNA, host gene, transcription, miRNA sponge, cancer

## Abstract

Circular RNAs (circRNAs) are a class of single-stranded, covalent closed-loop RNAs with tissue-/development-specific expression patterns. circRNAs are stable and play oncogenic or tumor suppressive roles in various aspects of cancer, including tumorigenesis, proliferation, apoptosis, metastasis, invasion, chemo-therapeutic resistance, and prognosis. circRNAs act as miRNA/protein sponges, protein scaffold, or template for translation. Increasing evidence shows circRNAs contribute to cancer progression *via* modulating the expression or function of their host genes. In this review, we summarize the latest progress in the regulation of host genes by circRNAs in human cancer. The works on circRNAs mediated regulation of host genes enhance us to understand the interaction between circRNAs and their host genes in human cancer.

## Introduction

Circular RNAs (circRNAs) have a history of nearly 30 years, initially considered as the outcome of transcriptional noise. circRNAs are a special class of noncoding RNAs (ncRNAs) derived from the back-splicing of pre-mRNAs or long non-coding RNAs (lncRNAs), and are more stable than linear host genes (1, 2). With the advances of deep sequencing and bioinformatics tools, a rapid increasing number of circRNAs have been identified in eukaryotic cells, and the expression profiles of circRNAs are tissue-/development-specific (3).

circRNAs gain increasing attention in the field of human cancer. The functions of circRNAs include adsorbing microRNAs (miRNAs) or proteins as sponges, regulating transcription or alternative splicing, interacting with RNA-binding proteins (RBPs), and translation (4, 5). Dysregulated circRNAs play oncogenic or tumor suppressive roles in various aspects of cancer, including cancer initiation, proliferation, apoptosis, metastasis, invasion, chemo-therapeutic resistance, and prognosis (6, 7). circRNAs may be diagnostic or therapeutic targets in human cancer (8, 9).

Accumulating evidence shows that circRNAs contribute to cancer progression *via* positively or negatively modulating the expression and function of their host genes (10–12). In this review, we summarize the latest progress in circRNA research on circRNA mediated regulation of host genes in human cancer.

## Regulatory Effects of circRNAs on Host Genes in Human Cancer

Back-splicing occurs coupled with the splicing of linear host RNAs, and may affect the splicing of linear RNAs (13). However, the efficiency of back-splicing is less than 1% of the canonical splicing (14). circRNAs can modulate the expression and function of their host genes *via* acting as miRNA sponges, regulating transcription, stability of mRNA, protein activity, and degradation. Moreover, circRNAs can regulate the expression of host genes by diverse regulatory effects or indirect pathways in human cancer.

### circRNAs Regulate Host Gene Expression as miRNA Sponges

circRNAs contain miRNA response elements (MREs) which bind to miRNAs and restrain their binding to the target mRNAs. Acting as miRNA sponges is the most extensively studied functions of circRNAs. Several circRNAs act as oncogenes in human cancer involving proliferation, migration, invasion, metastasis, glycolysis, and apoptosis by regulating the expression of their host genes *via* acting as miRNA sponges. Chen et al. found that circFNTA (hsa_circ_0084171), containing exon 2 to 6 of the *FNTA* gene, was increased in bladder cancer tissues from 41 patients as compared with adjacent para-tumor tissues. circFNTA promoted bladder cancer metastases and reduced cisplatin chemo-sensitivity by sponging miR-370, and the host gene of circFNTA *FNTA* was the downstream target of miR-370. Their work indicates that circRNAs may act as miRNA sponge to regulate the expression of their host genes, leading to bladder cancer metastasis and chemo-resistance (15). He et al. applied circRNA microarray and qPCR to screen and validate differently expressed circRNAs in triple negative breast cancer (TNBC) cell lines. circGFRA1 (hsa_circ_0005239) was increased in both TNBC cell lines and patients. High expression of circGFRA1 was correlated with poorer survival in 222 TNBC patients. circGFRA1 promoted proliferation and inhibited apoptosis by binding to miR-34a, relieving GFRA1 (16). Lung adenocarcinoma (LUAD) is one of the predominant reasons of cancer-related mortality. Enolase 1 (ENO1) is a glycolysis enzyme for glucose metabolism, and contributes to tumor progression. Zhou et al. carried out circRNA sequencing to identify differently expressed circRNAs in LUAD, and circENO1 (hsa_circ_0000013) was validated to be upregulated in LUAD tissues and cell lines. Mechanistically, circENO1 induced glycolysis of LUAD cells by acting as competing endogenous RNA (ceRNA) of miR-22-3p to promote the expression of *ENO1 (* 17).

On the other hand, several circRNAs act as tumor suppressors in human cancer *via* regulating the expression of their host genes through sponging miRNAs. Lv et al. applied RNA sequencing and qPCR to validate that circEPB41L5 was decreased in 49 glioblastoma tissues compared with six normal brain tissues. Low expression of circEPB41L5 was correlated to poor overall survival (OS) and progression-free survival (PFS). circEPB41L5 was derived from exons 17–25 of the *EPB41L5* gene. *In vitro* and *in vivo* experiments showed that circEPB41L5 inhibited proliferation, migration, clone formation, and invasion of glioblastoma by targeting the miR-19a/EPB41L5 axis (18).

These studies revealed that circRNAs may act as oncogene or tumor suppressor to regulate cancer growth, metabolism, apoptosis, metastasis, and drug-resistance by sponging miRNAs to release or inhibit the expression of their host genes (**Table 1**).

**Table 1:** circRNAs regulate host gene expression as miRNA sponges in human cancer.

circRNA	Cancer	Change	miRNA	Effect	Ref
circFNTA (hsa_circ_0084171)	Bladder cancer	up	miR-370-3p	oncogenic	(15)
circVANGL1	Bladder cancer	up	miR-605-3p	oncogenic	(19)
circITCH	Breast cancer	down	miR-7/-214	suppressive	(20)
circGFRA1 (hsa_circ_0005239)	Breast cancer	up	miR-34a	oncogenic	(16)
circAMOTL1 (hsa_circ_0004214)	Cervical cancer	up	miR-485-5p	oncogenic	(21)
circEPB41L5	Glioblastoma	down	miR-19a	suppressive	(18)
circITCH	Glioma	down	miR-214	suppressive	(22)
circSMO742	Glioma	up	miR-338-3p	oncogenic	(23)
circFLNA (hsa_circ_0092012)	LSCC	up	miR-486-3p	oncogenic	(24)
circENO1 (hsa_circ_0000013)	LUAD	up	miR-22-3p	oncogenic	(17)
circITCH	Lung cancer	down	miR-7/-214	suppressive	(25)
circLARP4	Ovarian cancer	down	miR-513b-5p	suppressive	(26)

LSCC, laryngeal squamous cell carcinoma; LUAD, lung adenocarcinoma.

### circRNAs Regulate the Transcription of Host Genes

circRNAs are able to regulate transcription and splicing in human cancer by recruiting proteins to the specific regions of target genes or sponging proteins to prevent their binding. Proteins include RBPs, transcription factors, DNA demethylase, and DNA methyltransferase. Promoter regions are the most widely studied specific regions for transcription regulation. circRNAs can positively or negatively regulate host gene transcription. Feng et al. screened circRNAs involved in prostate cancer (PC) progression using circRNA microarray. circXIAP (hsa_circ_0005276), derived from three exons of *XIAP*, and its host gene *XIAP* were both upregulated in PC. circXIAP was located in both cytoplasm and nucleus. circXIAP recruited FUS binding protein (FUS) to the promoter region of *XIAP* gene in nucleus and activated the transcription of *XIAP* so as to promote the proliferation, migration, and epithelial-mesenchymal transition (EMT) of PC cells (27). *FECR1* was a novel circRNA consisting of *FLI1* exons 4-2-3, bound to the promoter of *FLI1* and recruited DNA demethylase TET1 to induce DNA demethylation, increasing *FLI1* expression and promoting invasion of breast cancer cells (11). circCUX1 (hsa_circ_0132813), consisting of exon 2 and partial intron 2 of *CUX1*, was localized in the nucleus and increased in neuroblastoma. circCUX1 interacted with EWS RNA-binding protein 1 (EWSR1) and facilitated EWSR1-mediated transactivation of MYC-associated zinc finger protein (MAZ), promoting the transcriptional of CUX1, glycolysis and neuroblastoma progression (28).

circRNAs may also suppress cancer progression *via* impairing the transcription of their host genes through sponging RBP. circHuR (hsa_circ_0049027), containing exon 3 to 5 of *HuR*, was predominantly localized in the nucleus, and was decreased in gastric cancer (GC). circHuR interacted with CCHC-type zinc finger nucleic acid binding protein (CNBP), and restrained its binding to the promoter region of HuR, resulting in transcriptional repression of *HuR* and GC progression (29).

Exonic circRNAs (EcRNAs) were considered to mainly localize in the cytoplasm (30), while intronic circRNAs (CiRNAs) predominantly functioned in the nucleus (31). However, these mentioned EcRNAs were also distributed in the nucleus and regulated the transcription of their host genes (11, 27, 28). These recent studies suggest that EcRNAs not only functioned in cytoplasm, but also play pivotal roles in nucleus including regulating the transcription of their host genes (**Table 2**).

**Table 2:** circRNAs regulate host gene transcription in human cancer.

circRNA	Cancer	Change	Effect on host gene	Protein-interaction	Effect on cancer	Ref
circXIAP (hsa_circ_0005276)	Prostate cancer	up	promotive	FUS	oncogenic	(27)
FECR1	Breast cancer	–	promotive	TET1	oncogenic	(11)
circCUX1 (hsa_circ_0132813)	Neuroblastoma	up	promotive	EWSR1	oncogenic	(28)
circHuR (hsa_circ_0049027)	Gastric cancer	down	suppressive	CNBP	suppressive	(29)

### circRNAs Regulate the mRNA Stability of Host Genes

Beside of transcriptional regulation of host genes, circRNAs can also regulate the expression of host genes post-transcriptionally through affecting mRNA stability. circRNAs regulate the stability of host gene mRNA *via* recruiting or sponging RBPs which could bind with mRNA to enhance mRNA stability or induce mRNA instability. circE2F3 (hsa_circ_0075804), localized in cytoplasm, was upregulated in retinoblastoma, and overexpression of circE2F3 promoted the proliferation of retinoblastoma by increasing *E2F3* expression. Heterogeneous nuclear ribonucleoprotein K (HNRNPK) was able to bind with target mRNA and enhance mRNA stability. circE2F3 bound with HNRNPK and facilitate HNRNPK-mediated mRNA stability of *E2F3*, improving the stability of *E2F3* mRNA in retinoblastoma (32).

On the other hand, circRNAs may also induce mRNA instability of their host genes to suppress the expression of host genes. HuR is an extensively studied RBP which is able to bind with a wide range of RNAs, leading to translational promotion or suppression of target mRNAs through elongating or shortening the half-life of target mRNAs. By using RNA immunoprecipitation (RIP) and circRNA microarray, Abdelmohsen et al. screened HuR-interacted circRNAs in HeLa cells. circPABPN1 (hsa_circ_0031288) was the most highly enriched HuR-interacted circRNA. circPABPN1 competitively bound with HuR to disrupt the binding between HuR and PABPN1 mRNA, leading to instability of *PABPN1* mRNA and translational suppression of its host gene in Hela cells (33).

The mentioned circRNAs mainly regulated the translation of host genes in cytoplasm. Interestingly, circRNAs may play this regulatory role in nucleus. AUF1 is an AU-rich RBP which can shuttle between cytoplasm and nucleus. AUF1 could bind to the 3′-untranslated region (3′-UTR) of *DNMT1* mRNA and promote its degradation. circDNMT1 interacted with AUF1 and facilitated the nuclear translocation of AUF1, relieving DNMT1 mRNA from AUF1 induced instability of DNMT1 mRNA in breast cancer (34).

By binding with RBPs which are able to promote or suppress mRNA degradation, circRNAs may regulate the expression of their host genes in both cytoplasm and nucleus (**Table 3**).

**Table 3:** circRNAs regulate the stability of host gene mRNA in human cancer.

circRNA	Cancer	Change	Effect on host gene	Protein-interaction	Effect on cancer	Ref
circE2F3 (hsa_circ_0075804)	Retinoblastoma	up	promotive	HNRNPK	oncogenic	(32)
circDNMT1	Breast cancer	up	promotive	AUF1	oncogenic	(34)
circPABPN1 (hsa_circ_0031288)	Cervical cancer	–	suppressive	HuR	suppressive	(33)

### circRNAs Regulate the Activity and Degradation of Parental Proteins

Post-translational modifications are essential for the activity and degradation of proteins, such as acetylation, ubiquitination, sumoylation, and peptidyl-prolyl isomerization. Wnt signaling pathway contributes to the carcinogenesis of many malignancies, and GSK3β is a negative mediator of this pathway by phosphorylating Wnt and inducing ubiquitination-mediated degradation of Wnt. Hu et al. found that circGSK3β (hsa_circ_0007986) was localized in the cytoplasm and increased in esophageal squamous cell carcinoma (ESCC). Increased circGSK3β was positively correlated with advanced clinical stage and poor patient prognosis of ESCC. By using RIP and mass spectrometry, GSK3β, the parental protein of circGSK3β, was found to bind with circGSK3β in ESCC cell lines. circGSK3β bound to the N-terminal of GSK3β directly and inhibited its phosphorylation on Wnt, augmenting β-catenin signaling in ESCC (35). MDM2 is an E3 ubiquitin ligase and promotes tumor progression by targeting tumor suppressor proteins for ubiquitination mediated degradation, such as p53. Du et al. found that circFOXO3 acted as a protein scaffold recruiting both MDM2 and p53 to form MDM2-p53 complex *via* binding with the RING-finger domain of MDM2 and C-terminal regulatory domain of p53 respectively. circFOXO3 induced MDM2-mediated ubiquitination and degradation of p53 released FOXO3 from MDM2 induced ubiquitination and degradation, promoting the progression of breast cancer (36). These studies suggest that circRNAs may regulate the activity and degradation of parental protein by direct interacting with parental protein or disrupting the post-translational modification and subsequential degradation.

### Diverse Regulatory Effects of circRNAs on Host Gene in Human Cancer

circRNAs could regulate the expression of host genes *via* diverse pathways simultaneously. circCCND1 (hsa_circ_0023303) was increased in LSCC, and closely associated with adverse prognosis. circCCND1 bound to HuR and enhanced the binding of HuR to the 3′-UTR of *CCND1* mRNA to stabilize *CCND1* mRNA. On the other hand, circCCND1 sponged miR-646 to release *CCND1*. circCCND1 promoted the progression of LSCC through increasing *CCND1* expression by increasing its mRNA stability and disrupting miR-646 mediated suppression (37). AUF1 could bind to the 3′-UTR of target mRNA and induce mRNA degradation (34). circMMP9 (hsa_circ_0001162) was upregulated in oral squamous cell carcinoma (OSCC), and positively correlated with advanced TNM stage. circMMP9 simultaneously interacted with AUF1 and miR-149 to alleviate their inhibitory effect on *MMP9* 3′-UTR, resulting in enhanced *MMP9* expression and facilitating OSCC metastasis (38). circFBXW7 (hsa_circ_0001451) was decreased in TNBC, and was able to encode a novel 185aa peptide named FBXW7-185aa. FBXW7-185aa peptide bound to the deubiquitinating enzyme USP28 and prevented USP28 from binding to FBXW7, increasing the abundance of FBXW7. Moreover, circFBXW7 increased *FBXW7* expression *via* serving as a sponge of miR-197-3p to inhibit malignant progression of TNBC (39).

circRNAs may also regulate host gene expression *via* different mechanisms in different types of cancer. circSHPRH (hsa_circ_0001649) was decreased in both glioblastoma and hepatocellular carcinoma (HCC). circSHPRH inhibited HCC progression *via* acting as a ceRNA to sponge miR-127-5p, miR-612 and miR-4688 to relieve SHPRH (40). In glioblastoma, circSHPRH encoded a peptide named SHPRH-146aa. SHPRH-146aa competitively interacted with E3 ligase DTL, protecting full-length SHPRH from ubiquitination by DTL. Full-length SHPRH then suppressed glioblastoma progression by inducing proliferating cell nuclear antigen (PCNA) ubiquitination as an E3 ligase (10).

These studies support the cancer specific and complicated regulation of circRNAs on their host genes. This regulation of host genes by circRNAs may involve diverse mechanisms of circRNAs and may also be different in different types of cancer (**Table 4**).

**Table 4:** Diverse regulatory effects of circRNAs on host gene in human cancer.

circRNA	Cancer	Change	Effect on host gene	Protein-interaction	Pathway	Effect on cancer	Ref
circCCND1 (hsa_circ_0023303)	LSCC	up	promotive	HuR	circCCND1/HuR/CCND1 circCCND1/miR-646/CCND1	oncogenic	(37)
circMMP9 (hsa_circ_0001162)	OSCC	up	promotive	AUF1	circMMP9/miR-149/MMP9 circMMP/AUF1/MMP9	oncogenic	(38)
circFBXW7 (hsa_circ_0001451)	Breast cancer	down	promotive	USP28	circFBXW7/miR-197-3p/FBXW7 circFBXW7/peptide/USP28/FBXW7	suppressive	(39)

OSCC, oral squamous cell carcinoma.

## Conclusion

A number of host genes of circRNAs play oncogenic or suppressive role in tumorigenesis. circRNAs are derived from these genes and regulate the transcription, translation, parental protein activity and degradation of their host genes. Similar with the regulatory effects of circRNAs on other targets, circRNAs regulate host gene expression mainly *via* acting as miRNA sponges in human cancer. In addition, circRNAs may recruit or sponge some proteins to enhance or retard the transcription and mRNA stability of host genes. circRNAs can directly bind with host protein as protein sponge, or recruit proteins to regulate post-translational modification such as ubiquitination. Moreover, the regulation of host genes by circRNAs in cancer may involve a variety of classical mechanisms of circRNAs, and the suppressive mechanisms of circRNAs on host genes may be also different in different types of cancer, indicating the complicated regulatory effects of circRNAs on their host genes. The works on circRNAs mediated regulation of host genes enhance us to understand the interaction between circRNAs and their host genes in human cancer.

## Author Contributions

LC and YL designed this review. XW and HL collected the related paper. XW wrote the manuscript. All authors contributed to the article and approved the submitted version.

## Funding

This work was supported by grants from the National Natural Science Foundation of China (81572071 and 81000331).

## Conflict of Interest

The authors declare that the research was conducted in the absence of any commercial or financial relationships that could be construed as a potential conflict of interest.

## Abbreviations

circRNA, circular RNA; lncRNA, long non-coding RNA; miRNA, microRNA; RBP, RNA-binding protein; 3’-UTR, 3’-untranslated region; MRE, miRNA response element; TNBC, triple negative breast cancer; LUAD, lung adenocarcinoma; EMT, epithelial-mesenchymal transition; GC, gastric cancer; HCC, hepatocellular carcinoma; LSCC, laryngeal squamous cell carcinoma; ESCC, esophageal squamous cell carcinoma; OSCC, oral squamous cell carcinoma; PC, prostate cancer; RIP, RNA immunoprecipitation.
